# Entraining Alpha Activity Using Visual Stimulation in Patients With Chronic Musculoskeletal Pain: A Feasibility Study

**DOI:** 10.3389/fnins.2020.00828

**Published:** 2020-08-20

**Authors:** Laura J. Arendsen, James Henshaw, Christopher A. Brown, Manoj Sivan, Jason R. Taylor, Nelson J. Trujillo-Barreto, Alexander J. Casson, Anthony K. P. Jones

**Affiliations:** ^1^Division of Functional and Restorative Neurosurgery, Eberhart Karls University of Tübingen, Tübingen, Germany; ^2^Human Pain Research Group, Division of Neuroscience and Experimental Psychology, University of Manchester, Manchester, United Kingdom; ^3^Department of Psychological Sciences, University of Liverpool, Liverpool, United Kingdom; ^4^Leeds Institute of Rheumatology and Musculoskeletal Medicine, University of Leeds, Leeds, United Kingdom; ^5^Department of Electrical and Electronic Engineering, University of Manchester, Manchester, United Kingdom

**Keywords:** alpha activity, chronic pain, electroencephalography, entrainment, visual stimulation

## Abstract

Entraining alpha activity with rhythmic visual, auditory, and electrical stimulation can reduce experimentally induced pain. However, evidence for alpha entrainment and pain reduction in patients with chronic pain is limited. This feasibility study investigated whether visual alpha stimulation can increase alpha power in patients with chronic musculoskeletal pain and, secondarily, if chronic pain was reduced following stimulation. In a within-subject design, 20 patients underwent 4-min periods of stimulation at 10 Hz (alpha), 7 Hz (high-theta, control), and 1 Hz (control) in a pseudo-randomized order. Patients underwent stimulation both sitting and standing and verbally rated their pain before and after each stimulation block on a 0–10 numerical rating scale. Global alpha power was significantly higher during 10 Hz compared to 1 Hz stimulation when patients were standing (*t* = −6.08, *p* < 0.001). On a more regional level, a significant increase of alpha power was found for 10 Hz stimulation in the right-middle and left-posterior region when patients were sitting. With respect to our secondary aim, no significant reduction of pain intensity and unpleasantness was found. However, only the alpha stimulation resulted in a minimal clinically important difference in at least 50% of participants for pain intensity (50%) and unpleasantness ratings (65%) in the sitting condition. This study provides initial evidence for the potential of visual stimulation as a means to enhance alpha activity in patients with chronic musculoskeletal pain. The brief period of stimulation was insufficient to reduce chronic pain significantly. This study is the first to provide evidence that a brief period of visual stimulation at alpha frequency can significantly increase alpha power in patients with chronic musculoskeletal pain. A further larger study is warranted to investigate optimal dose and individual stimulation parameters to achieve pain relief in these patients.

## Introduction

Chronic pain is a prevalent and debilitating condition that has a wide-reaching impact on physical and mental well-being (Breivik et al., 2006; Van Hecke et al., 2013). Opioids and other medications are commonly prescribed to treat chronic pain (Turk et al., 2011). However, most medications have considerable side-effects and evidence for their long-term effectiveness is limited (Turk, 2002; Chou et al., 2015). In Europe, 40% of people with chronic pain report that their pain was inadequately managed (Breivik et al., 2006). Therefore, the development of alternative therapies to relieve pain is warranted.

Chronic musculoskeletal pain, regardless of the specific diagnostic classification, is influenced by multifactorial mechanisms including central sensitization, and is associated with changes in brain structure and function (Kulkarni et al., 2007; Gwilym et al., 2010; Baliki et al., 2014; Brown et al., 2014). These observations justify greater focus on the development of brain-based treatments that have generalizable efficacy across musculoskeletal conditions. A promising target for such treatments comes from evidence that chronic pain is associated with changes in oscillatory neural activity in the brain. Most commonly, an increase of theta power and beta power has been found (Sarnthein et al., 2006; Lim et al., 2016; Ploner et al., 2017), as well as a slowing of the peak alpha frequency that was found in patients with neurogenic pain, abdominal pain, and fibromyalgia (Sarnthein et al., 2006; Boord et al., 2008; De Vries et al., 2013; Lim et al., 2016). Thus, the brain’s response to pain provides a promising target for the development of novel pain therapies (Jensen et al., 2008).

A brain signal of particular interest as a therapeutic target is alpha activity, oscillatory neural activity in the frequency range of 8–12 Hz. Alpha activity gates the processing of incoming sensory information via a mechanism of functional inhibition (Jensen and Mazaheri, 2010). Incoming information is gated via the inhibition of brain regions processing irrelevant information (high alpha power), which routes the processing of information to task-relevant regions (low alpha power). This mechanism has been linked to top-down control and attention (Foxe and Snyder, 2011; Klimesch, 2012) and is also involved in pain processing. Alpha activity is decreased during experimental pain stimulation and has been found to encode subjective pain experience (Shao et al., 2012; Michail et al., 2016). Somatosensory alpha activity during pain (Hauck et al., 2015) and the anticipation of pain (May et al., 2012) is modulated by attention, and frontal alpha activity is increased following a placebo-induced expectation of pain relief (Huneke et al., 2013). Importantly, pre-stimulus somatosensory alpha power is inversely related to perceived pain: higher alpha power is associated with lower pain intensity and vice versa, both for experimental pain (Babiloni et al., 2006; Tu et al., 2016) and chronic pain (Camfferman et al., 2017; Ahn et al., 2019). Thus, neurotherapies that increase alpha power may have potential in reducing chronic pain.

Alpha activity can be enhanced through the application of rhythmic stimulation, including visual, auditory, and electrical stimulation (Thut et al., 2011). When presented with an external stimulation at a certain frequency, oscillatory neural activity at this same frequency synchronizes in phase with the external stimulation, a phenomenon often referred to as entrainment. This ultimately leads to an increase of power at the stimulation frequency at the population level (de Graaf et al., 2013; Helfrich et al., 2014; Spaak et al., 2014; Vossen et al., 2015).

Alpha entrainment has been successfully implemented to reduce experimentally induced pain using rhythmic visual (Ecsy et al., 2018), auditory (Ecsy et al., 2017), and transcranial alternating current stimulation (tACS) (Arendsen et al., 2018). However, to date, only one study successfully induced an increase in somatosensory alpha power that was correlated with a reduction in pain intensity in patients with chronic low-back pain (CLBP), using tACS at alpha frequency over somatosensory regions (Ahn et al., 2019). More work is required to understand the efficacy of alpha entrainment and the relationship between entrainment and analgesia across different clinical pain populations and using different stimulation modalities.

In particular, there is a lack of evidence that visual stimulation can entrain alpha activity in patients with varying diagnoses of chronic musculoskeletal pain. The effectiveness of external stimulation to entrain neural oscillations can be influenced by background activity in the brain (Feurra et al., 2013; Ruhnau et al., 2016; Gulbinaite et al., 2017) and an impairment of entrainment has been shown in patients with Parkinson’s disease (Te Woerd et al., 2017) and schizophrenia (Lakatos et al., 2013). Cerebral neuroplasticity in chronic pain may change the intrinsic or natural frequencies of affected areas of the brain, meaning that the gain (increased/decreased response) to external stimulation at a specific frequency might be changed, possibly to the extent of making entrainment to a specific frequency impossible. Therefore, evidence of alpha entrainment in chronic pain is critical in order to identify a treatment mechanism, should any analgesic effects follow from treatment, and is essential to justify the general application of alpha entrainment as a treatment.

This feasibility study primarily investigated whether visual alpha stimulation increased global alpha power in patients with chronic musculoskeletal pain. A secondary aim was to explore whether a brief period of alpha stimulation was also associated with reduced clinical pain. We used a within-subject design to compare alpha stimulation (10 Hz) to 1 Hz control stimulation. Furthermore, we also compared 7 Hz stimulation (high theta) to 1 Hz stimulation. An important confounding factor to take into account in the use of visual stimulation to entrain alpha activity is that synchronization of alpha oscillations can also be induced indirectly via the engagement of attentional mechanisms by the visual stimulus (Thut et al., 2011; Klimesch, 2012; Brüers and Vanrullen, 2018). Whereas 7 Hz visual stimulation could lead to indirect synchronization of alpha activity via attentional engagement similar to 10 Hz stimulation, it should not lead to direct alpha entrainment. Thus, the 7 Hz stimulation was included to address the confounding factor of attentional mechanisms related to the rhythmic visual stimulation. A correlation between alpha power and chronic pain intensity has been shown for frontal and somatosensory regions specifically (Tu et al., 2016; Ahn et al., 2019), suggesting that any reduction of chronic pain by alpha entrainment might be related to an increase of frontal and somatosensory alpha activity in particular. Therefore, this study also explored more regional changes in alpha power. Finally, this study also included a manipulation of the level of discomfort the patients experienced during stimulation, as previous studies have shown that background alpha activity (endogenous alpha) has an influence on alpha entrainment. Patients underwent stimulation both in a more uncomfortable condition (standing) and a resting condition (sitting). These different levels of discomfort might affect endogenous alpha activity, because chronic pain levels are negatively correlated with alpha power. In line with the finding that entraining alpha activity with tACS at alpha frequency is effective during a state of low endogenous alpha (eyes open), but to a lesser extent when endogenous alpha is high (eyes closed) (Neuling et al., 2013; Ruhnau et al., 2016), we might expect that visual alpha entrainment is more effective during the condition of stronger discomfort (lower endogenous alpha). However, it should be noted that another study found, in contrast, that higher levels of resting-state alpha power before stimulation were associated with more effective entrainment of alpha oscillations (Mathewson et al., 2012).

## Materials and Methods

### Participants

Twenty-two participants were recruited from local pain and musculoskeletal clinics (Salford Royal NHS Trust and North West CATS NHS) and support groups and from the University of Manchester. All participants gave written informed consent to take part in the study and received a reimbursement for their time and travel expenses. The study was approved by the North West-Liverpool East Research Ethics Committee (NHS Health Research Authority; reference number 17/NW/0255).

The inclusion criterion was a diagnosis of chronic musculoskeletal pain, i.e., presence of pain for at least 3 months. To promote generalizability of the findings, we did not focus on any particular diagnostic subgroup. Using opportunity sampling, the study resulted in the recruitment of 14 patients with fibromyalgia; 2 patients with osteoarthritis; 1 patient with fibromyalgia and osteoarthritis; 2 patients with low back pain; 1 patient with stenosis of the lower back; and 2 patients with widespread chronic pain (no specific diagnosis). All participants took part in a telephone interview to complete a screening questionnaire prior to participation to ensure they (1) were aged 18 or older; (2) did not have any difficulty understanding verbal or written English; (3) were not involved in any clinical trials at the time of testing; and (4) were not hospitalized/scheduled to be hospitalized during their participation in the study. To ensure that it was safe to undergo rhythmic visual stimulation, participants were excluded if they (1) were diagnosed with epilepsy or had ever had a convulsion or seizure; (2) had any first-degree relative with epilepsy; or (3) had ever experienced discomfort when exposed to flashing lights.

The datasets of two participants were removed from the final analysis as they were not able to complete the entire study due to high levels of pain and discomfort. These two participants both had a diagnosis of fibromyalgia. This resulted in a total of 20 participants who were included in the statistical analysis (mean age ± SD = 43.45 ± 16.82 years; 13 females) (Table 1). The employment status of these 20 participants was as follows: 6 were employed full-time; 6 were employed part-time; 4 were unemployed; 3 were retired; and 1 was a student. The annual income bracket for the participants was 6, £0–14.999; 10, £15.000–29.999; 1, £30.000–44.999; and 2, ≥60.000 (data missing for 1 participant).

**TABLE 1:** Demographic details for the 20 participants included in the statistical analysis of the study.

Patient number	Age	Gender	Pain condition	Pain history	HADS anxiety	HADS depression
1	25	F	FM	Diagnosed 5 years ago	13	7
3	66	M	FM and AO	Diagnosed >40 years ago	6	6
4	51	F	FM	Diagnosed 14 years ago	12	12
5	51	M	FM	Diagnosed 7 years ago	18	12
6	51	M	FM	Diagnosed 5 years ago	9	7
7	62	F	CLBP	–	5	8
8	50	F	OA	Diagnosed 9 years ago	9	4
9	26	F	FM	Diagnosed this year (2018), pain present >3 years	13	13
10	41	M	FM	Diagnosed this year (2018), symptoms started 10 years ago	9	10
12	19	F	CWP	Pain present for 3 years	6	1
13	56	F	FM	Diagnosed 4–5 years ago	8	6
14	71	M	Stenosis	Pain present >30 years	4	4
15	25	F	FM	Diagnosed this year (2018), pain present for 2 years	13	14
16	47	F	OA	–	12	5
17	46	F	FM	Diagnosed this year (2018), pain present for 4–5 years	9	13
18	70	M	CLBP	Pain present >2 years	2	2
19	23	F	FM	–	14	15
20	22	F	FM	Diagnosed 2 years ago	17	12
21	35	F	FM	Diagnosed this year (2018)	15	8
22	32	M	CWP	Pain present >9 years	8	8

*The pain history column contains a summary of the verbal response of the participants when asked to describe their pain history of as part of the screening procedure. The measurements were carried out in 2018. The HADS anxiety and HADS depression column provide the sum score for each sub scale of the Hospital Anxiety and Depression Scale (HADS). Each subscale contains seven items with sum scores ranging from 0 to 21. FM: fibromyalgia; AO: osteoarthritis; CLBP: chronic low back pain; CWP: chronic widespread pain, no specific diagnosis; F: female; M: male.*

### Visual Stimulation

**FIGURE 1:**
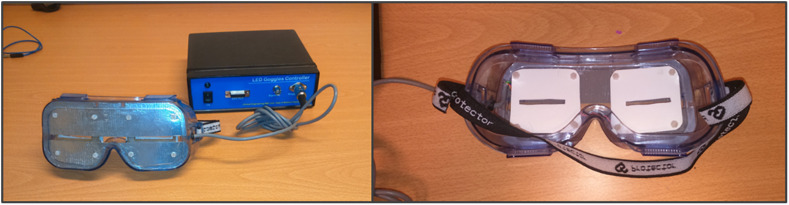
Goggles used for the visual stimulation at the three different stimulation frequencies (bespoke equipment—Medical Physics, Salford Royal NHS Foundation Trust). Eight LEDs were used in total, four around each eye. The goggles were kept in place with an elasticated headband.

All participants underwent visual stimulation at 10 Hz to entrain alpha activity and at the two control frequencies of 1 and 7 Hz. The visual stimulation was administered using goggles with eight LEDs, four around each eye (bespoke equipment—made by Medical Physics, Salford Royal NHS Foundation Trust; Figure 1). Rhythmic flashes were generated using bespoke software run in Matlab 2017a (The Mathworks, Inc., Natick, MA, United States; Matlab). Participants were asked to close their eyes during the stimulation, and brightness was adjusted for each individual participant to ensure that stimulation was administered at a comfortable brightness. After participants closed their eyes, they were presented with a 30-s 1-Hz stimulation sample to identify the brightness at which they could clearly perceive the visual stimulation without experiencing any discomfort.

**FIGURE 2:**
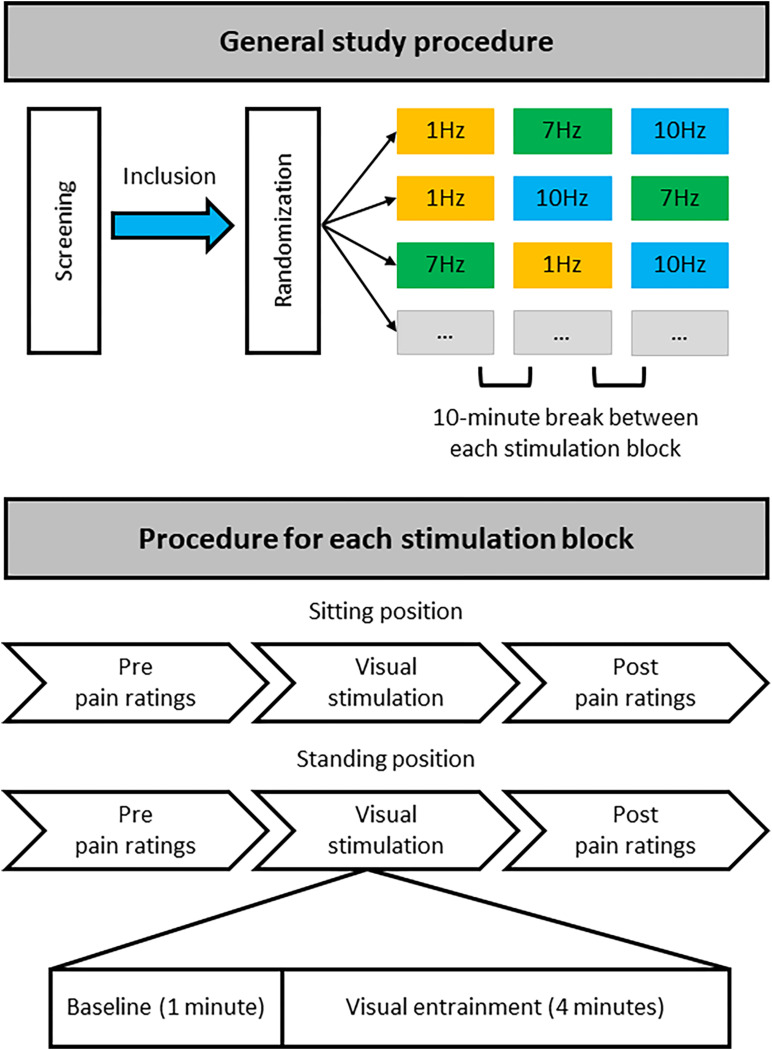
Overview of study procedure. During a single study visit each participant completed three stimulation blocks that each contained stimulation at one particular frequency (1, 7, or 10 Hz), with stimulation both in a sitting and standing position. For each stimulation block half of the participants always started with stimulation while sitting, the other half while standing. Each participant was randomly allocated to one of six possible stimulation frequency orders: 1, 7, 10 Hz; 1, 10, 7 Hz; 7, 1, 10 Hz; 7, 10, 1 Hz; 10, 1, 7 Hz; or 10, 7, 1 Hz. There was a break of at least 10 min between each stimulation block with a specific stimulation frequency to minimize any potential carryover effects. Thus, each participant received equal numbers of stimulation blocks at the different frequencies but randomized to a different order of stimulation blocks.

The three different stimulation frequencies were delivered in separate blocks (Figure 2). During each block a 1-min baseline period was followed by 4 min of rhythmic visual stimulation, while EEG was recorded. During the 1-min baseline, non-rhythmic visual stimulation was applied with a jittered interstimulus interval (ISI) between flashes. During this non-rhythmic stimulation period the signal phase would change by 180 degrees frequently, in a semi-random manner, so as not to cause any long-term entrainment effects. These phase changes would occur either every 1.6 s (50% of the time), every 1.15 s (25% of the time) or every 1.9 s (25% of the time). These non-rhythmic baseline periods before each stimulation period were later used for the EEG analysis to provide a standardized baseline for each stimulation condition. It was decided to include the non-rhythmic visual stimulation during the baseline period to ensure that the baseline and entrainment period were kept as similar as possible, e.g., with respect to luminance, with the only difference being that the stimulation during the baseline period was not rhythmic and would therefore not induce any direct alpha entrainment.

### Pain Assessment

To quantify chronic pain experience, participants were asked to verbally rate their pain intensity and pain unpleasantness using two 11-point numerical rating scales (NRS) ranging from 0 to 10 (0 = not at all intense/unpleasant, 10 = extremely intense/unpleasant).

To assess the effect of visual alpha stimulation on chronic pain, changes in chronic pain intensity and unpleasantness ratings were assessed in two settings associated with different levels of discomfort: (1) when participants were seated in a comfortable chair (sitting position); and (2) when participants were standing while holding the back of the chair for support (standing position). Participants were asked to rate their subjective level of discomfort as a result of the position they were in at the start and end of each stimulation block on an NRS ranging from 0 to 10 (0 = not at all uncomfortable, 10 = extremely uncomfortable). On average (mean ± SD) patients rated their discomfort at the start of each stimulation block as 3.94 ± 2.14 when sitting and as 4.78 ± 2.07 when standing. At the end of each stimulation block average discomfort was 3.80 ± 2.34 when patients were sitting and 5.53 ± 2.30 when standing. Stimulation was applied both in a setting of higher and lower discomfort to assess whether the level of ongoing discomfort might influence the effect of the visual stimulation.

Pain ratings were collected before and directly after each visual stimulation block, each block including a 1-min baseline and a 4-min entrainment period (at 1, 7, and 10 Hz), both in the sitting and standing condition (Figure 2).

### Questionnaires

Chronic pain and the outcome of chronic pain treatment are influenced by personality factors and pain-related cognition and beliefs (Keefe et al., 1989; Crombez et al., 1999; Granot and Ferber, 2005). Therefore, a series of questionnaires were included in this study to assess if the questionnaire variables were related to the effect of the visual alpha stimulation. This could potentially inform the design of a future, larger trial, e.g., whether to balance participants for these variables between treatment and control arms.

Participants were asked to complete a set of four questionnaires once, during the breaks between stimulation blocks: the Hospital Anxiety and Depression Scale (HADS) (Zigmond and Snaith, 1983); the Pain Self-Efficacy Questionnaire (PSEQ) (Nicholas, 1989); the Brief Pain Inventory (BPI) (Cleeland and Ryan, 1994); and the Multidimensional Health Locus of Control scale (MHLC) (Wallston et al., 1978).

Both anxiety and depression have been found to frequently co-occur with chronic pain conditions (Mcwilliams et al., 2003). Moreover, a positive association between pain experience and depression and anxiety has been found, both in an experimental pain setting (Walsh, 1998; Tang and Gibson, 2005) and a clinical pain setting (Geisser et al., 2000; Granot and Ferber, 2005). To assess anxiety and depression in the present study we used the HADS. The HADS is a valid self-assessment scale originally developed as a tool to reliably detect states of anxiety and depression in patients attending a general medical clinic (Zigmond and Snaith, 1983; Herrmann, 1997). The HADS comprises seven items to assess anxiety and seven items to assess depression. Participants are asked to tick the box that most closely represents how they were feeling in the past week on a 4-point scale ranging from 0 to 3. For example, “I feel tense or ‘wound up”’: (0) not at all; (1) occasionally; (2) a lot of the time; or (3) most of the time.

The BPI, PSEQ, and MHLC were used to gain further insight into the pain experience of the participants and their pain-related beliefs and cognitions.

The BPI is a tool to assess both pain intensity (sensory dimension) and pain interference (reactive dimension) in patients with chronic pain. The BPI was originally developed to assess cancer-related pain (Cleeland and Ryan, 1994) but is also a widely used and valid measure for patients with non-malignant chronic pain (Tan et al., 2004). Participants are asked to rate their worst and least pain intensity over the last 24 h, their average pain intensity, and their current pain intensity on a scale of 0 to 10 (sensory dimension). Participants are also asked to rate the degree to which pain interferes with seven domains of functioning on a scale of 0 to 10, for instance walking ability and relationships with other people (reactive dimension).

The PSEQ is a questionnaire designed to assess self-efficacy beliefs in people experiencing chronic pain (Nicholas, 1989), by assessing the confidence participants have in their ability to perform certain tasks and activities despite their pain. The PSEQ contains 10 items describing different settings/activities, such as “I can do most of the household chores (e.g., tidying-up, washing dishes, etc.), despite the pain” and “I can live a normal lifestyle, despite the pain.” Participants are asked to rate how confident they are that they can do these things at present despite the pain on a scale from 0 to 6, with 0 = not at all confident and 6 = completely confident.

The MHLC was developed to assess three dimensions of internal health locus of control, powerful others’ locus of control, and chance health of control (Wallston et al., 1978). The MHLC contains 18 items with a belief statement about the participant’s health, for example, “Whatever goes wrong with my pain condition is my own fault,” and “Other people play a big role in whether my pain condition improves, stays the same, or gets worse.” Participants are asked to rate to what extent they agree with each item on a scale of 1 to 6 (1 = strongly disagree and 6 = strongly agree).

### EEG Acquisition

An EEG was recorded during all visual stimulation blocks using 64 Ag/AgCl electrodes attached to a cap according to the extended standard 10–20 system, using the BrainCap MR, BrainAmp DC/MR amplifiers, and the EEG data recording software BrainVision Recorder (Brain Products GmbH, Germany). The FCz electrode was used as a reference electrode and AFz as the ground electrode. EEG was recorded with a sampling rate of 500 Hz and band-pass filter settings of DC-100 Hz.

### Procedure

All participants attended the lab for a single study visit during which they underwent visual stimulation at all 3 frequencies (1, 7, and 10 Hz), once while sitting down and once while standing up. This resulted in a total of 6 stimulation conditions: 3 visual stimulation frequencies (1, 7, 10 Hz) × 2 positions (sitting and standing). For each condition, a 1-min baseline period was followed by a 4-min stimulation period (Figure 2).

After obtaining written informed consent, completing the EEG setup, and identifying the individual stimulation brightness, each participant was pseudo-randomly allocated to 1 of the 6 possible stimulation frequency orders. All participants completed 3 stimulation blocks, with each block containing 1 specific stimulation frequency. Participants experienced each stimulation frequency both sitting and standing. Half of the participants completed each stimulation block in the sitting position first, and the other half started with the standing position first. Pain intensity and unpleasantness were assessed before and after each stimulation condition, i.e., directly before and after the stimulation in the sitting position and also directly before and after the stimulation in the standing position. After each stimulation block participants had a break of at least 10 min before carrying on with the next block, to limit potential carryover effects of the previous block of stimulation. Although both the experimenter and the participants were blinded to the order of visual stimulation frequencies, due to the nature of the stimulation (visual) the frequency of stimulation for each block became apparent as soon as the stimulation was started. However, importantly, participants were not provided with any clues as to which was the expected therapeutic condition.

### EEG Analysis

The EEG recordings were imported into Matlab (The Mathworks, Inc., Natick, MA, United States; Matlab version R2017a). A number of pre-processing and artifact removal steps were carried out on the continuous EEG data using the EEGLAB toolbox (Delorme and Makeig, 2004) in the following order: (1) interpolation of any bad channels (spherical interpolation); (2) re-referencing to the common average; (3) and high-pass (0.05 Hz) and low-pass filtering (30 Hz). A median of 0.5 channels were interpolated with a range from 0 to 5. Next, the continuous data were segmented into 2-s consecutive epochs to accommodate later visual inspection of the data post-independent component analysis (ICA). Finally, as an EEG for each of the different stimulation conditions was recorded and saved in separate files, the data from the different stimulation conditions were combined into one single data file per participant. These data were decomposed into independent signals using ICA in order to remove components reflecting artifactual sources, with components from frontal sources reflecting eyeblinks and eye movements selected for removal. The number of ICs to be calculated was adjusted for the number of interpolated channels (*N* channels – *N* interpolated channels). The median number of components removed was 2.5 with a range of 1 to 6. The reconstructed EEG data were then visually inspected to remove any remaining muscle artifacts and any other remaining large artifacts, i.e., large spikes and jumps present in the EEG data (on average 4.79% of trials were removed per participant).

Frequency analysis was performed using the Fieldtrip Toolbox (Oostenveld et al., 2011). Average alpha power (8–12 Hz) was calculated for each visual stimulation condition (1, 7, and 10 Hz) using FFT with a single Hanning taper and non-overlapping windows. All individual alpha power outcomes were log-transformed.

**FIGURE 3:**
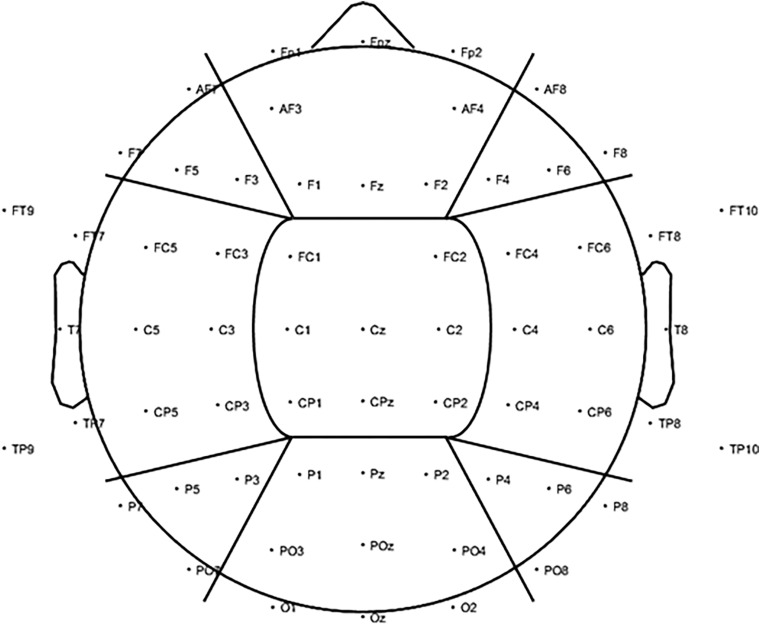
To investigate changes in alpha power (8–12 Hz) for the visual alpha stimulation regionally, further analysis was carried out based on nine ROIs. We included three anterior ROIs: left anterior (LA), including electrodes AF7, F7, F5, and F3; central anterior (CA), including electrodes FP1, FPz, FP2, AF3, AF4, F1, Fz, and F2; and right anterior (RA): including electrodes AF8, F4, F6, and F8. Three middle ROIs: left middle (LM), including electrodes FT9, FT7, FC5, FC3, T7, C5, C3, TP9, TP7, CP5, and CP3; central middle (CM), including electrodes FC1, FC2, C1, Cz, C2, CP1, CPz, and CP2; and right middle (RM): including electrodes FC4, FC6, FT8, FT10, C4, C6, T8, CP4, CP6, TP8, and TP10. Finally, three posterior ROIs: left posterior (LP), including electrodes P7, P5, P3, and PO7; central posterior (CP), including electrodes P1, Pz, P2, PO3, POz, PO4, O1, Oz, and O2; and right posterior (RP): including electrodes P4, P6, P8, and PO8.

Global alpha power was calculated by averaging the log-transformed alpha power from 8–12 Hz across all 2-s epochs per condition across all electrodes, resulting in a single average alpha power outcome per visual stimulation condition. The same was applied to the baseline periods preceding each stimulation condition. Next, the average log-transformed alpha power during each stimulation condition was standardized against its respective baseline period (subtraction method: log alpha power entrainment – log alpha power baseline). To assess changes in alpha activity on a more regional level, alpha power was also calculated for nine regions of interest (ROIs) by averaging over the electrodes in each region only (Figure 3).

### Statistical Analysis

Statistical analysis was performed using SPSS version 22 (IBM Corp, Armonk, NY, United States). To assess the effect of the visual stimulation on global alpha power, i.e., alpha power averaged across all electrodes, a repeated-measures ANOVA with the factors stimulation (1, 7, and 10 Hz) and position (sitting and standing) was applied.

Next, to further explore the effect of alpha stimulation on a more regional level, changes in alpha power were also compared for the nine ROIs (Figure 3) using a repeated-measures ANOVA with the factors stimulation (1, 7, and 10 Hz), position (sitting and standing), the left-to-right (L-R) ROI factor (left, central, and right), and the anterior-to-posterior (A-P) ROI (anterior, middle, and posterior).

Finally, two repeated-measures ANOVAs with the factors stimulation (1, 7, and 10 Hz), position (sitting and standing), and time (pre- and post-stimulation) were applied to assess a change in pain intensity and unpleasantness ratings respectively.

For all ANOVAs, in the case of a violation of sphericity, Greenhouse–Geisser corrected outcomes were used. To correct for multiple comparisons, the Bonferroni correction was applied.

### Minimal Clinically Important Difference in Pain Ratings

In line with the recommendations of the Initiative on Methods, Measurement, and Pain Assessment in Clinical Trials (IMMPACT) consensus statement (Dworkin et al., 2005), we also assessed what percentage of participants showed a minimally important clinical difference (MCID) in pain intensity and unpleasantness. A MCID is considered the smallest difference in pain rating that patients perceive as important and is reflected by a 15% reduction in pain intensity/unpleasantness rating compared to baseline [(pain rating post-stimulation − pain rating pre-stimulation)/pain rating pre-stimulation] (Dworkin et al., 2008).

### Correlations

To assess the potential relationship between alpha activity and changes in pain due to the alpha stimulation, correlations between standardized global alpha power and the change in pain intensity/unpleasantness rating were calculated. In detail, the average log-transformed alpha power during 10 Hz stimulation standardized against its baseline period was used (subtraction method: alpha power entrainment – alpha power baseline). To calculate the change in the pain intensity/unpleasantness ratings we used the formula ratings post-stimulation – ratings pre-stimulation. Thus, a negative correlation would reflect that higher alpha power during stimulation (compared to baseline) was associated with lower pain ratings post-stimulation and vice versa.

To assess the relationship between changes in pain following alpha stimulation and personality factors and pain-related cognitions and beliefs, correlations between the different questionnaire scores and the change in pain intensity and unpleasantness rating were calculated. The following questionnaire outcomes were used: the sum score for the depression subscale and anxiety subscale separately (HADS); the individual rating for average, worst, and least pain intensity over the last 24 h and a sum score for the seven pain interference items (BPI); a single sum score for all PSEQ items; and a separate sum score for each of the three subscales of the MHLC.

## Results

### Global Alpha Power

**FIGURE 4:**
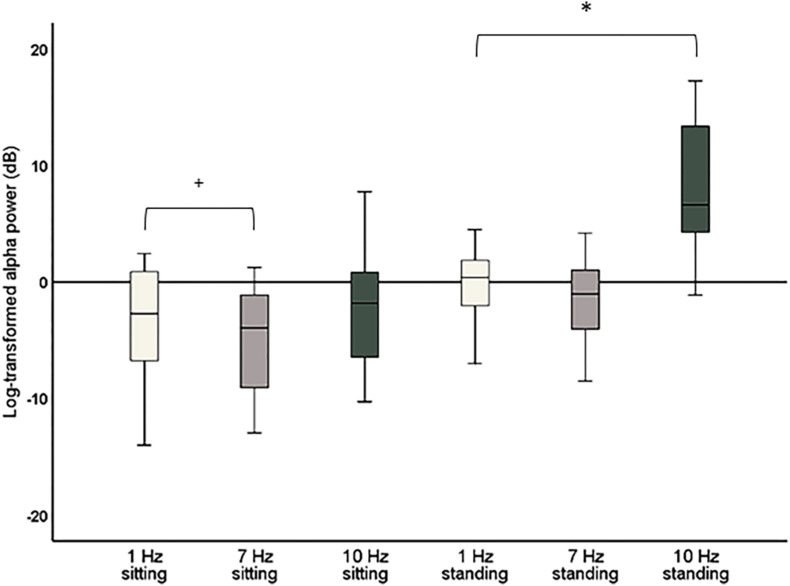
Boxplots of global alpha power (8–12 Hz) during 1 Hz, 7 Hz, and 10 Hz visual stimulation standardized against their respective baseline period, i.e., the change in global alpha power during stimulation compared to baseline. Significant effects after correction for multiple comparisons are marked with *. Effects that did not survive corrections for multiple comparisons but had a *p* < 0.05 are marked with **^+^**.

The repeated-measures ANOVA with the factors Stimulation (1, 7, and 10 Hz) and Position (sitting and standing) showed a significant main effect of Stimulation (*F*_2_,_38_ = 34.88; *p* < 0.001; partial η^2^ = 0.65) on global alpha power and a significant interaction between Stimulation and Position (*F*_2_,_38_ = 13.48; *p* < 0.001; partial η^2^ = 0.42; Figure 4). *Post hoc* repeated-measures *t*-tests showed that alpha power was significantly higher during 10 Hz stimulation compared to the 1 Hz control stimulation during the standing condition (*t* = −6.08, *p* < 0.001). There was no significant increase of global alpha power during the sitting condition (*t* = −1.30, *p* = 0.21). No increase of alpha power was found for the 7 Hz condition compared to 1 Hz condition, only a significant decrease of global alpha power for 7 Hz compared to 1 Hz stimulation in the sitting condition (*t* = 2.51, *p* = 0.021). However, this effect did not survive correction of multiple comparisons (corrected significance level of 0.0125). Finally, alpha power was also significantly higher during 10 Hz stimulation compared to 7 Hz stimulation, both for the sitting condition (*t* = −3.69, *p* = 0.002) and the standing condition (*t* = −6.03, *p* < 0.001).

As a significant increase of alpha power for 10 Hz compared to 1 Hz stimulation was only found for the standing condition, a further *post hoc* repeated-measures *t*-test was calculated to assess whether there was a significant difference in global alpha power comparing sitting and standing for the 10 Hz stimulation. This *t*-test showed that global alpha power was significantly higher during the 10 Hz stimulation in the standing condition (*t* = −6.40, *p* < 0.001).

### ROI Alpha Power

A repeated-measures ANOVA was calculated to assess more regional changes in alpha power with the factors Stimulation (1, 7, and 10 Hz), Position (sitting and standing), L-R ROI (left, central, and right) and A-P ROI (anterior, middle, and posterior). A significant main effect was found for the A-P ROI factor (*F*_1_._34_,_25_._49_ = 11.43; *p* = 0.001; partial η^2^ = 0.38; Greenhouse–Geisser corrected). Moreover, a significant interaction between Stimulation, Position, and A-P ROI was found (*F*_2_._48_,_47_._07_ = 6.65; *p* < 0.002; partial η^2^ = 0.26; Greenhouse–Geisser corrected). Similarly, a significant main effect was found for the L-R ROI factor (*F*_1_._25_,_23_._76_ = 4.50; *p* = 0.037; partial η^2^ = 0.19; Greenhouse–Geisser corrected), accompanied by a significant interaction between Stimulation and L-R ROI (*F*_1_._25_,_23_._68_ = 11.87; *p* = 0.001; partial η^2^ = 0.39; Greenhouse–Geisser corrected) and a significant interaction between Stimulation, Position, and the L-R ROI (*F*_1_._91_,_36_._20_ = 12.24; *p* < 0.001; partial η^2^ = 0.39; Greenhouse–Geisser corrected). Thus, the effect of visual stimulation on alpha power was different depending on position and on scalp region.

**FIGURE 5:**
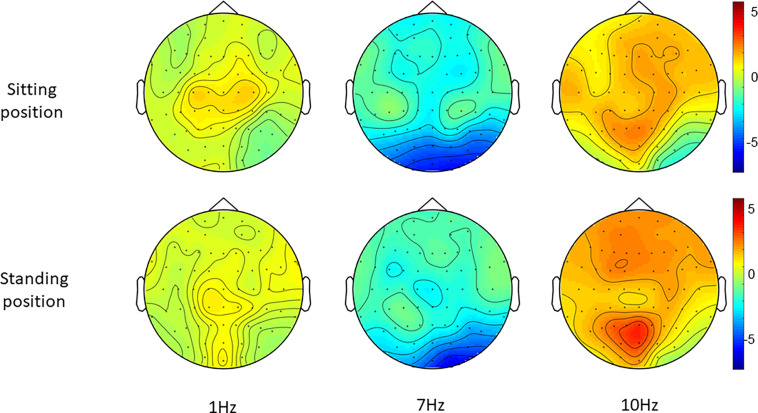
Topographies of standardized alpha power (8–12 Hz), i.e., the change in alpha power during each entrainment condition compared to their respective baseline period.

*Post hoc* repeated-measures *t*-tests showed that alpha power was significantly higher during 10 Hz stimulation compared to 1 Hz stimulation in the right-middle region (RM) and the left-posterior region (LP) in particular when participants were sitting (Tables 2, 4 and Figure 5). In the standing condition, alpha power was higher during 10 Hz compared to 1 Hz stimulation across a wider range of mostly middle-anterior regions; however, these effects did not survive correction for multiple comparisons (corrected significance level of 0.0056).

**TABLE 2:** Outcomes of the *post hoc* repeated measures *t*-tests comparing 1 and 10 Hz stimulation for the nine ROIs separately.

ROI	Sitting	Standing
Left-anterior (LA)	*t* = −1.66, *p* = 0.11	*t* = −2.43, *p* = 0.025
Central-anterior (CA)	*t* = −1.51, *p* = 0.15	*t* = −2.22, *p* = 0.039
Right-anterior (RA)	*t* = −1.96, *p* = 0.065	*t* = −2.26, *p* = 0.036
Left-middle (LM)	*t* = −1.93, *p* = 0.069	*t* = −2.34, *p* = 0.031
Central-middle (CM)	*t* = −1.08, *p* = 0.29	*t* = −1.23, *p* = 0.24
Right-middle (RM)	*t* = −4.44, *p* < 0.001*	*t* = −1.62, *p* = 0.12
Left-posterior (LP)	*t* = −4.33, *p* < 0.001*	*t* = −2.32, *p* = 0.032
Central-posterior (CP)	*t* = −1.19, *p* = 0.25	*t* = −1.66, *p* = 0.11
Right-posterior (RP)	*t* = −0.61, *p* = 0.55	*t* = −1.04, *p* = 0.31

*Significant *t*-tests are marked with a ^∗^ (Bonferroni-corrected significance level).*

*Post hoc t*-tests comparing 7 and 1 Hz visual stimulation showed that alpha power was significantly lower during 7 Hz stimulation compared to 1 Hz stimulation, in particular in the central-middle region (CM) and the central-posterior region (CP; Tables 3, 4 and Figure 5), when participants were sitting. A similar pattern was present when participants were standing; however, this did not survive correction for multiple comparisons for the central-middle region.

**TABLE 3:** Outcomes of the *post hoc* repeated measures *t*-tests comparing 1 and 7 Hz stimulation for the nine ROIs.

ROI	Sitting	Standing
Left-anterior (LA)	*t* = 2.44, *p* = 0.025	*t* = 2.01, *p* = 0.059
Central-anterior (CA)	*t* = 3.08, *p* = 0.006	*t* = 1.73, *p* = 0.099
Right-anterior (RA)	*t* = 2.70, *p* = 0.014	*t* = 1.93, *p* = 0.068
Left-middle (LM)	*t* = 2.87, *p* = 0.010	*t* = 2.23, *p* = 0.038
Central-middle (CM)	*t* = 3.66, *p* = 0.002*	*t* = 3.03, *p* = 0.007
Right-middle (RM)	*t* = 2.76, *p* = 0.012	*t* = 2.57, *p* = 0.019
Left-posterior (LP)	*t* = −1.82, *p* = 0.085	*t* = 1.99, *p* = 0.062
Central-posterior (CP)	*t* = 4.44, *p* < 0.001*	*t* = 3.45, *p* = 0.003*
Right-posterior (RP)	*t* = 2.66, *p* = 0.016	*t* = 2.73, *p* = 0.013

*Significant *t*-tests are marked with a ^∗^ for the Bonferroni-corrected significance level.*

**TABLE 4:** Standardized alpha power (dB) per stimulation condition (1, 7, and 10 Hz) for each ROI and for sitting (top) and standing (bottom) separately.

ROI	1 Hz	7 Hz	10 Hz
**Sitting**			
Left-anterior (LA)	0.43 (2.46)	−0.92 (1.90)	1.38 (2.33)
Central-anterior (CA)	0.81 (2.75)	−1.10 (2.08)	1.80 (2.71)
Right-anterior (RA)	0.70 (2.73)	−1.04 (2.06)	1.91 (2.35)
Left-middle (LM)	0.83 (2.01)	−0.50 (1.99)	1.57 (1.95)
Central-middle (CM)	1.35 (2.38)	−1.09 (2.49)	1.87 (2.62)
Right-middle (RM)	0.84 (2.07)	−0.82 (2.12)	7.13 (5.15)
Left-posterior (LP)	−4.65 (8.36)	−1.63 (2.66)	1.55 (3.35)
Central-posterior (CP)	0.68 (2.94)	−2.48 (2.33)	1.56 (3.14)
Right-posterior (RP)	−0.016 (3.25)	−2.17 (2.88)	0.38 (2.92)
**Standing**			
Left-anterior (LA)	0.75 (1.78)	−0.74 (2.45)	2.22 (1.91)
Central-anterior (CA)	0.79 (1.75)	−0.53 (2.62)	2.32 (2.43)
Right-anterior (RA)	0.82 (1.59)	−0.54 (2.33)	2.16 (2.42)
Left-middle (LM)	0.67 (1.69)	−0.69 (2.26)	1.83 (1.66)
Central-middle (CM)	1.12 (1.78)	−1.01 (2.59)	1.86 (2.13)
Right-middle (RM)	0.91 (1.77)	−0.74 (2.31)	1.75 (1.59)
Left-posterior (LP)	0.55 (1.69)	−0.99 (2.96)	2.21 (2.81)
Central-posterior (CP)	0.95 (1.76)	−1.88 (2.87)	2.36 (2.95)
Right-posterior (RP)	0.45 (2.12)	−2.14 (3.55)	1.26 (2.88)

### Intensity Ratings

**FIGURE 6:**
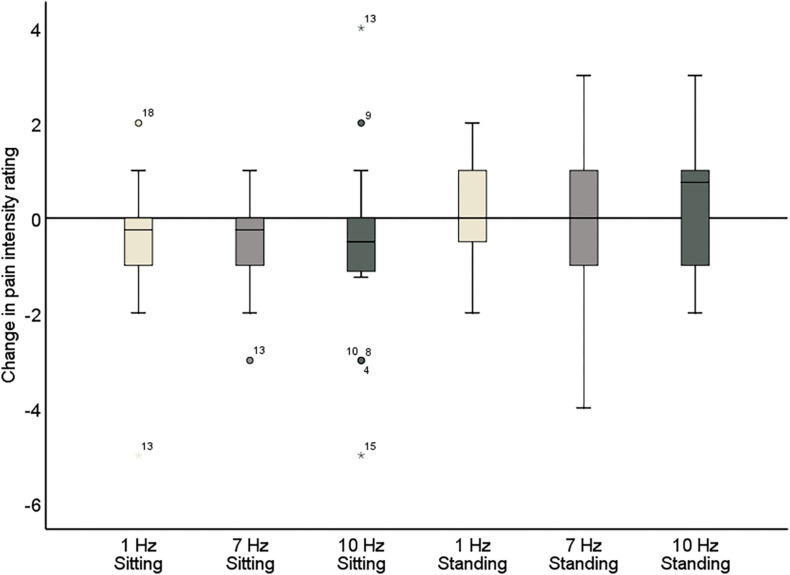
Change in pain intensity ratings comparing pre- and post-stimulation for 1, 7, and 10 Hz stimulation, both in the sitting and standing condition. A negative score reflects a reduction in pain and a positive score reflects an increase of pain following the stimulation.

The repeated-measures ANOVA demonstrated a significant main effect of Position (sitting and standing) on intensity ratings (*F*_1_,_19_ = 12.32; *p* = 0.002; partial η^2^ = 0.39), but no significant main effect of Stimulation (*F*_2_,_38_ = 1.80; *p* = 0.18; partial η^2^ = 0.087). There was a significant interaction between Stimulation and Time (pre- and post-stimulation) (*F*_1_._46_,_27_._80_ = 0.065; *p* = 0.89; partial η^2^ = 0.003; Greenhouse–Geisser corrected), nor a significant interaction between Stimulation, Position, and Time (*F*_2_,_38_ = 0.59; *p* = 0.56; partial η^2^ = 0.030) (Table 5 and Figure 6). Moreover, the repeated-measures *t*-tests further assessing an effect on pain intensity for the alpha stimulation (10 Hz), specifically, did not find a significant change in pain intensity ratings comparing pre- and post-alpha stimulation (sitting: *t* = 1.54, *p* = 0.14; and standing: *t* = −1.11, *p* = 0.28).

**TABLE 5:** Pain intensity ratings (Mean ± SD) pre- and post-stimulation, for the 1, 7, and 10 Hz stimulation condition and for the sitting and standing positions.

Intensity ratings				
Frequency	Sitting pre	Sitting post	Standing pre	Standing post
1 Hz	4.70 ± 1.95	4.15 ± 2.20	5.18 ± 1.84	5.28 ± 1.97
7 Hz	4.43 ± 1.70	4.03 ± 2.07	4.75 ± 1.99	4.60 ± 2.47
10 Hz	4.39 ± 2.12	3.73 ± 2.01	4.45 ± 1.87	4.78 ± 2.07

### Unpleasantness Ratings

**FIGURE 7:**
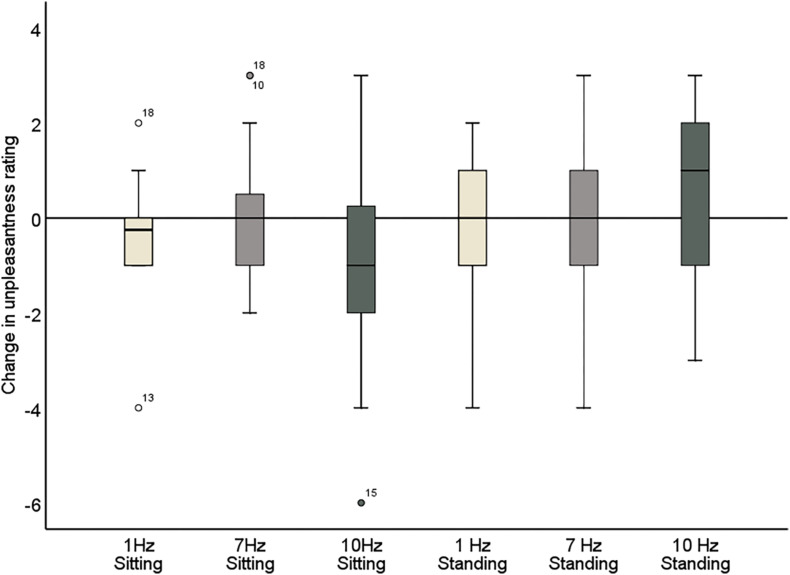
Change in pain unpleasantness ratings comparing pre- and post-stimulation for 1, 7, and 10 Hz stimulation, in both the sitting and standing conditions. A negative score reflects a reduction in pain and a positive score reflects an increase of pain following the stimulation.

The repeated measures ANOVA demonstrated a significant main effect of Position (sitting and standing) on unpleasantness ratings (*F*_1_,_19_ = 12.61; *p* = 0.002; partial η^2^ = 0.40), but no significant main effect of Stimulation (*F*_2_,_38_ = 1.78; *p* = 0.18; partial η^2^ = 0.085). There was also not a significant interaction between Stimulation and Time (*F*_2_,_38_ = 0.73; *p* = 0.49; partial η^2^ = 0.037), nor a significant interaction between Stimulation, Position, and Time (*F*_2_,_38_ = 2.63; *p* = 0.085; partial η^2^ = 0.12) (Table 6 and Figure 7). Moreover, the repeated-measures *t*-tests assessing an effect on pain unpleasantness for the alpha stimulation (10 Hz) specifically did not find a significant change of pain unpleasantness ratings comparing pre- and post-alpha stimulation (sitting: *t* = 1.77, *p* = 0.093; and standing: *t* = −1.32, *p* = 0.20).

**TABLE 6:** Pain unpleasantness ratings (Mean ± SD) pre- and post-stimulation, for the 1, 7, and 10 Hz stimulation condition and for the sitting and standing positions.

Unpleasantness ratings				
Frequency	Sitting pre	Sitting post	Standing pre	Standing post
1 Hz	4.55 ± 2.04	4.10 ± 2.37	5.40 ± 1.98	5.20 ± 2.02
7 Hz	4.20 ± 1.82	4.33 ± 2.34	4.63 ± 1.75	4.65 ± 2.24
10 Hz	4.38 ± 2.31	3.50 ± 2.07	4.28 ± 1.89	4.80 ± 2.17

### Minimal Clinically Important Difference in Pain Ratings

#### Sitting Condition

We assessed the number of participants that showed a MCID (percentage change >15%) in pain ratings, for the three different stimulation conditions separately. For the intensity ratings, 50% of participants demonstrated a MCID for the 10 Hz stimulation. A similar value was found for the 1 Hz condition (45%). For the 7 Hz condition an MCID was found for 35%. For the unpleasantness ratings, 65% of participants demonstrated an MCID for the 10 Hz stimulation. This percentage was lower for the 1 Hz condition (40%) and the 7 Hz condition (30%). Only the alpha stimulation led to an MCID in pain intensity and unpleasantness for ≥50% of participants.

#### Standing Condition

Next, we assessed the number of participants who showed an MCID (percentage change >15%) in pain ratings, for the three different stimulation conditions separately when the participants were standing. For the intensity ratings, 25% of participants demonstrated an MCID (percentage change >15%) for the 10 Hz stimulation. A similar value was found for the 1 Hz condition (20%) and the 7 Hz condition (30%). For the unpleasantness ratings, 30% of participants demonstrated an MCID for the 10 Hz stimulation. This percentage was also found for the 1 Hz condition (30%) and the 7 Hz condition (30%). None of the stimulation conditions led to an MCID in pain intensity or unpleasantness for ≥50% of participants in the standing condition.

### Correlations

The correlations between standardized global alpha power during 10 Hz stimulation (log alpha power during 10 Hz stimulation – baseline log alpha power) and the difference in intensity/unpleasantness ratings comparing pre- and post-stimulation were calculated (ratings post-stimulation – ratings pre-stimulation) (Table 7). No significant correlation was found for the intensity ratings (sitting: *r* = 0.34; *p* = 0.14; *N* = 20; standing: *r* = 0.16; *p* = 0.51; *N* = 20). For the unpleasantness ratings no significant correlation with global alpha power was found either for the standing condition (*r* = 0.11; *p* = 0.65; *N* = 20). A correlation was found for the sitting condition (*r* = 0.46; *p* = 0.04; *N* = 20); however, this did not survive correction for multiple comparisons (corrected significance level = 0.0125).

**TABLE 7:** Overview of the results of the correlation analysis.

	Intensity ratings		Unpleasantness ratings	
	Sitting	Standing	Sitting	Standing
Global alpha	*r* = 0.34; *p* = 0.14	*r* = 0.16; *p* = 0.51	*r* = 0.46; *p* = 0.04**^+^**	*r* = 0.11; *p* = 0.65
RM-alpha	*r* = −0.40; *p* = 0.082		*r* = −0.41; *p* = 0.073	
LP-alpha	*r* = 0.43; *p* = 0.060		*r* = 0.54; *p* = 0.015**^+^**	
HADS-A	*r* = 0.048; *p* = 0.84	*r* = −0.11; *p* = 0.64	*r* = 0.10; *p* = 0.68	*r* = −0.038; *p* = 0.87
HADS-D	*r* = 0.081; *p* = 0.73	*r* = 0.30; *p* = 0.20	*r* = 0.12; *p* = 0.60	*r* = 0.50; *p* = 0.026**^+^**
BPI-average	*r* = −0.18; *p* = 0.47	*r* = −0.042; *p* = 0.87	*r* = −0.041; *p* = 0.87	*r* = 0.13; *p* = 0.60
BPI-worst	*r* = −0.25; *p* = 0.30	*r* = 0.15; *p* = 0.54	*r* = −0.057; *p* = 0.82	*r* = 0.19; *p* = 0.43
BPI-least	*r* = 0.11; *p* = 0.65	*r* = −0.29; *p* = 0.23	*r* = 0.20; *p* = 0.41	*r* = −0.23; *p* = 0.35
BPI-I	*r* = −0.18; *p* = 0.45	*r* = 0.21; *p* = 0.39	*r* = −0.12; *p* = 0.63	*r* = 0.32; *p* = 0.18
PSEQ	*r* = −0.055; *p* = 0.82	*r* = −0.35; *p* = 0.14	*r* = 0.040; *p* = 0.87	*r* = −0.40; *p* = 0.077
MHLC-I	*r* = 0.19; *p* = 0.43	*r* = −0.014; *p* = 0.96	*r* = 0.23; *p* = 0.34	*r* = 0.077; *p* = 0.75
MHLC-O	*r* = 0.090; *p* = 0.71	*r* = 0.14; *p* = 0.56	*r* = −0.036; *p* = 0.88	*r* = 0.097; *p* = 0.68
MHLC-C	*r* = −0.41; *p* = 0.075	*r* = −0.040; *p* = 0.87	*r* = -0.29; *p* = 0.21	*r* = −0.020; *p* = 0.93

*Correlations were calculated for the change in pain intensity/unpleasantness rating and standardized global alpha power (Global alpha). *Post hoc* correlations were also calculated for the two ROIs that showed significantly higher alpha power during 10 Hz stimulation compared to 1 Hz stimulation for the sitting condition only (RM-alpha and LP-alpha). Secondly, correlations were calculated between the change in pain intensity/unpleasantness rating and a number of questionnaire outcomes: the sum score of the HADS anxiety (HADS-A) and HADS depression subscale (HADS-D); the BPI average, worst, and least pain intensity rating (BPI-average, BPI-worst, and BPI-least) and the sum score for the seven pain interference items (BPI-I); the sum score for all PSEQ items (PSEQ); and a sum score for each of the three subscales of the MHLC, internal health locus of control, powerful others locus of control, and chance health of control (MHLC-I, MHLC-O, and MHLC-C). Significant correlations after correction for multiple comparisons are marked with ^∗^. Correlations that did not survive corrections for multiple comparisons but had a *p* < 0.05 are marked with **^+^**.*

*Post hoc* it was decided also to explore the correlations between the change in pain intensity/unpleasantness ratings and standardized alpha power for the two ROIs that showed a significant increase of alpha power during 10 Hz stimulation compared to 1 Hz stimulation in the sitting condition, the right-middle (RM) and left-posterior (LP) ROI (Table 7). For the RM ROI, there was no significant correlation between change in pain ratings (ratings post-stimulation – ratings pre-stimulation) and alpha power (intensity ratings: *r* = −0.40; *p* = 0.082; *N* = 20; unpleasantness ratings: *r* = −0.41; *p* = 0.073; *N* = 20). For the LP ROI, no significant correlation was found either for the intensity ratings (*r* = 0.43; *p* = 0.060; *N* = 20). A correlation was identified for the unpleasantness ratings (*r* = 0.54; *p* = 0.015; *N* = 20), but this did not survive correction for multiple comparisons (corrected significance level = 0.0125).

Finally, correlations were assessed between the change in intensity/unpleasantness ratings and the questionnaire outcomes (Table 7). No significant correlation between ratings and any of the questionnaire outcomes was found. A correlation between pain unpleasantness ratings and the HADS Depression subscale was identified in the standing condition (*r* = 0.50; *p* = 0.026; *N* = 20). However, this did not survive correction for multiple comparisons.

## Discussion

Emerging evidence shows an inverse relationship between alpha power and chronic pain (Camfferman et al., 2017; Ahn et al., 2019). Therefore, alpha activity has been proposed as a key target for novel neuromodulatory therapies to manage chronic pain (Jensen et al., 2008). This feasibility study primarily aimed to assess the efficacy of visual alpha stimulation to enhance alpha activity in patients with chronic musculoskeletal pain. Secondarily, it was evaluated whether a brief period of alpha stimulation was also sufficient to reduce chronic pain. The main finding of this study was that visual alpha stimulation can effectively enhance alpha activity in patients with chronic musculoskeletal pain. Global alpha power was significantly higher during alpha stimulation compared to the 1 Hz control stimulation when patients were experiencing stronger discomfort (standing condition). On a more regional level, a significant increase of alpha activity was also found in the right-middle and left-posterior region when patients were sitting. With respect to our secondary aim, 4 min of alpha stimulation was not sufficient to significantly reduce chronic pain. However, only the alpha stimulation resulted in an MCID in at least 50% of participants for the pain intensity (50%) and unpleasantness ratings (65%). This study is the first to demonstrate the efficacy of rhythmic visual stimulation to modulate alpha activity in patients with chronic pain. However, further study is warranted to investigate the optimal dose and individual stimulation parameters (Krause and Cohen Kadosh, 2014), such as duration and frequency of entrainment to achieve significant pain relief.

Whereas both 7 and 10 Hz stimulation can result in an indirect entrainment of alpha activity via attentional mechanisms (Thut et al., 2011), only the 10 Hz stimulation should lead to a direct entrainment of alpha. No evidence for entrainment of alpha activity during 7 Hz stimulation was found, i.e., global alpha power was not significantly higher during 7 Hz stimulation compared to 1 Hz stimulation. Only a significant decrease of alpha power was found for 7 Hz stimulation compared to 1 Hz stimulation in central-middle and central-posterior regions. In addition, global alpha power was significantly higher during 10 Hz stimulation compared to 7 Hz stimulation, both for the sitting condition and the standing condition. Together, this suggests that the effect of alpha (10 Hz) stimulation on alpha power found in this study is likely the result of direct entrainment, and does not only reflect a non-specific effect of attention being directed away from the pain by visual stimulation.

The present study’s findings build on the findings by Ecsy et al. (2018), who previously demonstrated that visual alpha stimulation can increase alpha power and reduce pain in an experimental pain setting, albeit in healthy individuals experiencing acute laser pain rather than in patients with chronic pain. Qualitatively, when we compare the analyses of regional changes, the scalp regions showing increases in alpha activity are similar between the two studies, with a posterior dominance in both cases and weaker (in the current study, statistically non-significant) evidence for additional fronto-central increases. In the absence of a more robust quantitative comparison between patients and healthy controls (which would require a further controlled study), we cannot conclude that alpha entrainment differs in patients with chronic musculoskeletal pain, but we cannot rule out this possibility either.

Ahn et al. (2019) provided the first evidence that alpha stimulation can be used successfully in a clinical pain setting. They demonstrated that alpha tACS applied over somatosensory regions enhances somatosensory alpha power in patients with CLBP. Here we demonstrate that rhythmic visual stimulation can also modulate alpha activity in patients. Moreover, as this study included patients with various chronic musculoskeletal pain conditions, it also offers a first indication that the modulation of alpha activity with alpha stimulation can be generalized across different chronic pain populations.

The effect of visual alpha stimulation may be influenced by the level of discomfort experienced by the patients. Only when a patient was standing—a setting of stronger discomfort possibly related to lower endogenous alpha—did stimulation result in a global entrainment of alpha activity. In addition, global alpha power was significantly higher during standing compared to sitting during the 10 Hz stimulation. This would be in line with previous studies showing that alpha entrainment with tACS at alpha frequency is most effective when endogenous alpha is low (Neuling et al., 2013; Ruhnau et al., 2016). When the patient was sitting (lower discomfort), the stimulation did not result in a significant increase of global alpha power. However, a significant increase was found for two regions of interest, suggesting a more regional entrainment of alpha activity. Previously, a negative correlation has been found between somatosensory alpha power and perceived pain intensity for experimentally induced pain (Babiloni et al., 2006; Tu et al., 2016) and between frontal and somatosensory alpha power and chronic pain intensity (Camfferman et al., 2017). Ahn et al. (2019) also found that the increase of frontal and somatosensory alpha power by alpha tACS was associated with pain relief. In the present study, increasing global alpha power with visual stimulation did not result in a significant reduction of pain intensity and unpleasantness. Moreover, the present study found only a non-significant negative correlation between standardized somatosensory alpha power (right-middle ROI) and the change in pain intensity (*r* = −0.40; *p* = 0.082) and unpleasantness (*r* = −0.41; *p* = 0.073) following alpha stimulation (sitting condition). As these correlations were only marginally significant and based on a relatively small sample (*N* = 20), no confident conclusions can be drawn from these findings. However, where this study only included brief periods of stimulation, Ahn et al. (2019) applied alpha tACS for 40 min. In an experimental pain setting with pain-free volunteers, Ecsy et al. (2017, 2018) achieved a significant reduction in pain ratings using 10 min of auditory and visual stimulation and Arendsen et al. (2018) applied alpha tACS for 15–20 min. This feasibility study focused primarily on the entrainment of alpha activity, where it has been shown that even very short periods of stimulation can entrain alpha oscillations (Herrmann, 2001; Mathewson et al., 2012; Notbohm and Herrmann, 2016). However, to also reduce chronic pain, longer stimulation periods might be required. Moreover, this feasibility study included a small and heterogenous group of patients with chronic musculoskeletal pain, which introduces the possibility that the study is simply underpowered to find an effect of the stimulation on chronic pain. Further investigation with a larger sample size is needed to confirm whether a longer period of visual alpha stimulation leads to a significant reduction of chronic pain.

Further inspection of the individual changes in pain intensity and unpleasantness in response to the alpha stimulation showed that a wide variability in pain response was present (Figure 7 and Tables 5, 6). Whereas some patients showed a reduction of several points on the 11-point NRS, others did not improve at all or even showed an increase of pain. Large variability in response is a problem for neurostimulation techniques in general. To improve the efficacy of neurostimulation interventions to manage chronic pain, it is important to take into account inter- and intra-individual factors such as cognitive, psychological, and neurophysiological state, and methodological factors that might contribute to this variability (Li et al., 2015; Fertonani and Miniussi, 2017). In this study we did not identify a relationship between patient characteristics (as assessed with the questionnaires) and the pain response. However, larger sample sizes (e.g., 80–100) are likely needed for such analyses to be adequately powered for medium effect sizes. Another important source of variability in the effects of neurostimulation is brain-state dependency, i.e., the effect of neurostimulation depends on the timing of stimulation with respect to the underlying brain state. A number of studies have shown that applying neurostimulation in a brain-state dependent manner can enhance the modulation of corticospinal excitability (Saito et al., 2013; Kaneko et al., 2014; Kraus et al., 2016). Ultimately, taking into account these factors in the application of neurostimulation should lead to a more personalized and adaptive neuromodulatory therapy to reduce chronic pain.

Evidence shows that the efficacy of alpha entrainment depends on the distance between the stimulation frequency and the individual alpha peak frequency (IAF) (Herrmann et al., 2016; Notbohm et al., 2016; Gulbinaite et al., 2017). Thus, tailoring the frequency of the visual alpha stimulation to each individual could improve the effect of alpha stimulation in patients with chronic pain. Moreover, recent studies have also explored the potential of combined stimulation. Anodal tDCS over the primary motor cortex (M1) combined with peripheral electrical stimulation led to an enhanced, long-lasting, and clinically important reduction in chronic pain (Boggio et al., 2009; Schabrun et al., 2014; Hazime et al., 2017). Together, these recent developments in the application of neurostimulation offer promising future directions for application of alpha stimulation to reduce chronic pain.

To successfully implement visual alpha stimulation to reduce chronic pain, it is also important to better understand the relationship between alpha activity and chronic pain. So far, most studies have focused on the role of alpha activity in the perception of experimentally induced pain in pain-free individuals. Although there are some initial findings showing a negative correlation between frontal and somatosensory alpha power and chronic pain (Camfferman et al., 2017; Ahn et al., 2019), the functional role of alpha activity in the perception of chronic pain remains unclear. Experimental pain studies have demonstrated that the relationship between alpha activity and pain is influenced by attention (May et al., 2012; Hauck et al., 2015) and expectations about pain (Huneke et al., 2013; Arendsen et al., 2018), and that pain expectations can influence the effect of neuro-stimulation on pain perception (Arendsen et al., 2018). However, the relationship between attention, expectation, and alpha activity in a setting of chronic pain is little understood. It is important to better understand how these factors influence the relationship between alpha activity and chronic pain and the effectiveness of alpha stimulation to reduce chronic pain.

The present study showed that visual alpha stimulation offers a means to modulate alpha activity in patients with chronic pain in a lab-based environment. Whereas this is an important first step, further development is required to transform this lab-based application into a therapeutic technique that patients can use in their own home with therapeutic benefit. In a parallel study (Locke et al., 2020), a first qualitative assessment of a smartphone-based alpha entrainment technology was carried out. Individuals with chronic pain were asked about their experience with using the technology at home, using a virtual-reality headset for rhythmic visual stimulation and headphones for rhythmic auditory stimulation (binaural beats). The study provided initial support for the acceptability and usability of this smartphone-based technology as an affordable and accessible alternative to manage chronic pain. An important next step is to investigate the effectiveness of longer periods and multiple sessions of alpha stimulation to reduce chronic pain in the lab and at home, to translate these initial findings into a technology that can effectively reduce pain in a home-based setting.

## Conclusion

To conclude, this study provides first evidence that visual stimulation at alpha frequency can be used to increase alpha power in patients with musculoskeletal pain. However, a brief 4-min period of stimulation was not sufficient to reduce chronic pain. This study is a first step in the development of a novel neurostimulation approach to reducing chronic pain. Further study is warranted to investigate individual stimulation and optimal dose parameters (Krause and Cohen Kadosh, 2014) to achieve significant pain relief in a larger group of patients. Together with the further development of a home-based neurostimulation platform, this could ultimately lead to the implementation of alpha stimulation as an affordable and accessible neurotherapy to manage chronic pain.

## Author’s Note

This manuscript has been released as a pre-print at BioRxiv (Arendsen et al., 2020).

## Data Availability Statement

The raw data supporting the conclusions of this article will be made available by the authors, without undue reservation.

## Ethics Statement

The studies involving human participants were reviewed and approved by the North West – Liverpool East Research Ethics Committee (NHS Health Research Authority; reference number 17/NW/0255). The patients/participants provided their written informed consent to participate in this study.

## Author Contributions

AC, AJ, CB, JH, JT, LA, MS, and NT-B: conceptualization, study design, and writing – review and editing. AJ, LA, and MS: recruitment. LA: data collection and writing – original draft preparation. CB, JH, and LA: analysis. All authors contributed to the article and approved the submitted version.

## Conflict of Interest

The authors declare that the research was conducted in the absence of any commercial or financial relationships that could be construed as a potential conflict of interest.
